# Effects of Cold Atmospheric Plasma (CAP) on ß-Defensins, Inflammatory Cytokines, and Apoptosis-Related Molecules in Keratinocytes *In Vitro* and *In Vivo*

**DOI:** 10.1371/journal.pone.0120041

**Published:** 2015-03-13

**Authors:** Stephanie Arndt, Michael Landthaler, Julia L. Zimmermann, Petra Unger, Eva Wacker, Tetsuji Shimizu, Yang-Fang Li, Gregor E. Morfill, Anja-Katrin Bosserhoff, Sigrid Karrer

**Affiliations:** 1 Institute of Pathology, University Regensburg, D-93042 Regensburg, Germany; 2 Department of Dermatology, University Hospital Regensburg, D-93042 Regensburg, Germany; 3 Max Planck Institute for Extraterrestrial Physics, D-85748 Garching, Germany; 4 Institute of Biochemistry and Molecular Medicine, University Erlangen, D-91054—Erlangen, Germany; University Hospital Schleswig-Holstein, Campus Kiel, GERMANY

## Abstract

Cold atmospheric plasma (CAP) has been gaining increasing interest as a new approach for the treatment of skin diseases or wounds. Although this approach has demonstrated promising antibacterial activity, its exact mechanism of action remains unclear. This study explored *in vitro* and *in vivo* whether CAP influences gene expression and molecular mechanisms in keratinocytes. Our results revealed that a 2 min CAP treatment using the MicroPlaSter ß in analogy to the performed clinical studies for wound treatment induces expression of IL-8, TGF-ß1, and TGF-ß2. *In vitro* and *in vivo* assays indicated that keratinocyte proliferation, migration, and apoptotic mechanisms were not affected by the CAP treatment under the applied conditions. Further, we observed that antimicrobial peptides of the ß-defensin family are upregulated after CAP treatment. In summary, our results suggest that a 2 min application of CAP induces gene expression of key regulators important for inflammation and wound healing without causing proliferation, migration or cell death in keratinocytes. The induction of ß-defensins in keratinocytes describes an absolutely new plasma strategy. Activation of antimicrobial peptides supports the well-known antibacterial effect of CAP treatment, whereas the mechanism of ß-defensin activation by CAP is not investigated so far.

## Introduction

Plasmas have been used for a long time for sterilization of medical equipment or for sterile packaging in the food industry [1–3]. In the last years, cold atmospheric plasma (CAP) sources have been developed that provide the possibility to extend the plasma treatment to living tissue. This opens up new horizons. CAP has the great potential for contact-free disinfection in seconds and can access even microscopic openings and ragged surfaces. In contrast to antibiotic or antiseptic treatments and according to present knowledge, allergic or toxic reactions are not expected by the CAP treatment, however, the molecular effects on eukaryotic cells have been insufficiently investigated up to now. Several *in vivo* and *ex vivo* studies with different plasma devices approved a rapid reduction of the bacterial load or even of candida biofilms after the CAP treatment [4–11].

Studies on the effects of CAP on mammalian cells have been also conducted by several research groups. Summarizing the *in vitro* results demonstrated that CAP affects cells in a dosage- and device-dependent manner and can cause necrosis, apoptosis, or cell detachment [12–16]. Besides the alteration of cell adhesion, exposure to CAP causes a temporary cell membrane permeabilization [17] and inhibition of cell migration [18]. In addition, authors reported an induction of cell proliferation after treatment with CAP. The observed stimulation of cell proliferation presumably results from the release of growth factors (e.g. fibroblast growth factor-2 (FGF2)) by the plasma-treated cells but seems to be again cell- and plasma device specific [19]. Our own recently published data on fibroblasts revealed that a 2 min CAP treatment using the MicroPlaSter ß has no influence on fibroblast proliferation, whereas fibroblast migration was significantly induced [20].

Comparing the results from different groups working in the area of plasma medicine is therefore not easy due to different plasma sources, treatment modalities and conditions. All the more it is indispensable to understand the molecular basis behind the plasma phenomenon.

Identification of target genes and molecular mechanisms which are affected by the CAP treatment in keratinocytes is the basis of the present study. Understanding the mechanisms of CAP-cell interactions is absolutely necessary and crucial to assure safety during CAP-disinfection or CAP-skin/wound treatment. Furthermore, the effect of CAP on cell culture studies with normal keratinocytes have not been investigated so far, although keratinocytes of the epidermal layer are the most directly affected cells during the CAP treatment of the skin. We chose the MicroPlaSter ß for our experiments, since several published clinical studies on humans with e.g. acute or chronic wounds already showed the beneficial effects of this specific plasma source [5, 6, 21–23].

## Materials and Methods

### Plasma device and treatment of cells

The CAP device employed in this study was the MicroPlaSter ß plasma torch system (Microwave 2.45 GHz, 80 W, argon flow 4.0 l/min, treatment diameter ~5cm) developed by the Max Planck Institute for Extraterrestrial Physics in Garching/Germany and built by ADTEC Plasma Technology Co. Ltd., Hiroshima/London. This device was also used in clinical studies for the treatment of chronic and acute wounds [5] and for skin pruritus [23]. Treatment of keratinocytes with CAP was performed in analogy to our previous study with fibroblasts and described in detail in our manuscript [20].

### Cell culture conditions

Primary human skin keratinocytes were purchased from Cascade Biologics (Eugene, OR, USA; Lot: 5C0733) and ATCC (Manassas, VA, USA; Lot: 58732331 and Lot: 60245430) and were cultured according to [24].

For the treatment of keratinocytes with cell culture supernatants, 100000 cells were seeded into each well of a 12-well plate and incubated with 1 ml of supernatant obtained from keratinocytes 24 h after cells were directly treated with CAP or control for 2 min. Supernatants were collected from 3 individual experiments. Gene expression was analyzed 6 h, 24 h, 48 h, and 72 h after incubation was started.

### Human Apoptosis Array

Human Apoptosis Arrays (array kit ARY009, R&D systems, Minneapolis, USA) were performed according to the manufacturer´s instructions and allow a parallel determination of 35 apoptosis related proteins (Bad, Bax, Bcl-2, Bcl-x, Pro-Caspase-3, Cleaved Caspase-3, Catalase, cIAP-1, cIAP-2, Clapsin, Clusterin, Cytochrome c, TRAIL R1/DR4, TRAIL R2/DR5, FADD, Fas/TNFSF6, HIF-1 alpha, HO-1/HMOX1/HSP32, HO-2/HMOX2, HSP27, HSP60, HSP70, HTRA2/Omi, Livin, PON2, p21/CIP/CDNK1A, p27/Kip1, phospho-p53 (S15), phospho-p53 (S46), phospho-p53 (S392), phospho-Rad17 (S635), SMAC/Diablo, Survivin, TNF RI/TNFRSF1A, XIAP). Keratinocyte treatment, isolation of cellular extracts, and densitometry of the array spots were performed according to [20].

### Human Cytokine Array

Human Cytokine Arrays (array kit ARY005, R&D systems, Minneapolis, USA) were performed according to [20] using cell supernatants, collected from keratinocytes 24 h after a 2 min CAP treatment or from untreated control cells and offer a parallel determination of 36 cytokines, chemokines, and acute phase proteins (C5a, CD40 ligand, G-CSF, GM-CSF, CXCL1/GRO alpha, CCL1/I-309, ICAM-1, IFN-gamma, IL-1 alpha, IL-1 beta, IL-1 ra, IL-2, IL-4, IL-5, IL-6, IL-8, IL-10, IL-12p70, IL-13, IL-16, IL-17, IL-17E, IL-23, IL-27, IL-32 alpha, CXCL10/IP-10, CXCL11/I-TAC, CCL2/MCP-1, MIF, CCL3/MIP-1 alpha, CCL4/MIP-1 beta, CCL5/RANTES, CXCL12/SDF-1, Serpin E1/PAI-1, TNF-alpha, TREM-1).

### Enzyme-linked immunosorbent assay (ELISA)

Cell supernatants were collected 24 h, 48 h, and 72 h after a 2 min CAP treatment or of untreated control keratinocytes and were analyzed by ELISAs, respectively, according to [20]. ELISAs for the detection of TGF-ß1 and TGF-ß2 were received from R&D Systems, Wiesbaden-Nordenstadt, Germany, and for the detection of human HBD-1, HBD-2, and HBD-3 were obtained from US Biological, Salem, MA, USA. Each sample was assayed in duplicates, and the entire experiments were performed three times.

### FlowCytomix Simplex Technology

The Human FlowCytomix Simplex Detection System (eBioscience, San Diego, CA 92121, USA) was used to quantify the protein amount of IL-8 from cell supernatants collected 24 h, 48 h, and 72 h after a 2 min CAP treatment or from untreated control keratinocytes according to [20]. Each sample was assayed in duplicates, and the entire experiments were performed three times.

### Analysis of mRNA expression by quantitative RT-PCR

Isolation of total cellular RNA from keratinocytes 6 h, 24 h, 48 h, and 72 h after the CAP treatment and reverse transcriptase reaction was performed according to [20] and [25]. Quantitative RT-PCR was performed with specific sets of primers and conditions (Table 1) applying LightCycler technology (Roche Diagnostics, Mannheim, Germany) as described [25]. Each RT-PCR was performed in duplicates with cDNA of at least three different keratinocyte cell cultures.

**Table 1:** Primers and conditions.

*Primer name*	*Forward primer*	*Reverse primer*	*Condition(annealing, melting)*
human ß-actin	5′-TACGTCGCCCTGGACTTCGAGC-3′	5′-GATGGAGCCGCCGATCCACACGG-3′	ann. 62°C, mt 78°C
human IL-8	5′-CTGCAGCTCTGTGTGAAGGTG-3′	5′-ACAGAGCTCTCTTCCATCAG-3′	ann. 60°C, mt 81°C
human TGF-ß1	5′-GCAGAGCTGCGTCTGCTGAGGC-3′	5′-CCCGTTGATGTCCACTTGCAGTG-3′	ann. 65°C, mt 85°C
human TGF-ß2	5′-TCTAGGGTGGAAATGGATACACGAACC-3′	5′-TGTTACAAGCATCATCGTTGTCGTCG-3′	ann. 65°C, mt 78°C
human BD-1 (HBD-1)	5′-TTGTCTGAGATGGCCTCAGGTGGTAAC-3′	5′-ATACTTCAAAAGCAATTTTCCTTTAT-3′	ann. 60°C, mt 82°C
human BD-2 (HBD-2)	5′- TCAGCCATGAGGGTCTTGTA-3′	5′-GGATCGCCTATACCACCAAA-3′	ann. 60°C, mt 78°C
human BD-3 (HBD-3)	5′-TTGCTCTTCCTGTTTTTGGTG-3′	5′-CGCCTCTGACTCTGCAATAA-3′	ann. 60°C, mt 74°C
murine ß-actin	5′-GTGGGCCGCTCTAGGCACCAA-3′	5′-CTCTTTGATGTCACGCACGATTTC-3′	ann. 60°C, mt 86°C
murine TGF-ß1 (mTGF-ß1)	5′-GGCTCTGGAGAACAGCACATC-3′	5´-CAAGCAGTCCTTCCCTTCAGG-3′	ann. 60°C, mt 89°C
murine TGF-ß2 (mTGF-ß2)	5′-TGGCGCTCAGTCTGTCTACCT-3′	5´-TTGGCGTAGTACTCCTCGTCG-3′	ann. 60°C, mt 89°C
murine BD-1 (MBD-1)	5′-CCAGATGGAGCCAGGTGTTG-3′	5′-TGGTATTAGATGGGCAGCTGG-3′	ann. 65°C, mt 83°C
murine BD-2 (MBD-2)	5′-GCTGCTGATATGCTGCCTCC-3′	5′-TGGCAGAAGGAGGACAAATGG-3′	ann. 63°C, mt 75°C
murine BD-3 (MBD-3)	5′-TTCTCCTGGTGCTGCTGTCTC-3′	5′-GAGTGTTGCCAATGCACCG-3′	ann. 63°C, mt 82°C

Primer sequence is given from 5´➔ 3´ DNA strand. Ann: annealing temperature; mt: melting temperature.

### Proliferation Assay (XTT)

XTT Proliferation Assay (Roche Diagnostics, Mannheim, Germany) with keratinocytes was performed 24 h, 48 h and 72 h after CAP exposure for 2 min according to [20, 26]. Each sample was assayed in duplicates, and the entire experiments were performed three times with three different keratinocyte cell cultures.

### Migration Assay

The migratory behaviour of CAP treated or control treated keratinocytes was assayed by means of a wound healing assay. The migration rate into the “wound area” was documented and measured 7 h and 12 h after the culture-insert was removed leaving a cell-free gap (“defined wound”) as described in detail in [20].

### Mouse experiments

For the experiments 129Sv/Ev female mice between 8 and 12 weeks of age were used. The mice were anesthetized according to [20], and animals´ backs were shaved. Four mice became a daily CAP treatment using the MicroPlaSter ß (80 W, argon flow 4 l/min) for 2 min, 5 days long. Control mice were treated with the placebo modus of the MicroPlaSter ß for 2 min instead of plasma, to resemble the same stress-situation for all animals. To circumvent any potential indirect activation of the innate immune system, we performed an antiseptic treatment with octenidine (Octenisept Schuelke & Mayr GmbH, Norderstedt, Germany) as described by [7] prior to each CAP or placebo treatment using additionally four mice per group. All mice were immediately sacrificed after therapy and the treated skin area was excised.

Preparation of epidermal RNA was performed according to [20] and mRNA expression was analyzed by quantitative RT-PCR as described above. All animal experiments were conducted with appropriate permission from the animal rights commission of the state of Bavaria and maintained in agreement with the European Union guidelines. All experiments were approved by the Committee on the Ethics of Animal Experiments of the University of Regensburg, Germany (Permit Number: 54–2532.1–10/11).

### Immunohistochemistry

Immunohistochemical staining was performed on 4-μm formalin-fixed, paraffin-embedded skin sections. Endogenous peroxidase activity was blocked with 3% hydrogen peroxide (Dako Cytomation GmbH, Hamburg, Germany). Antigen retrieval was carried out in Tris-EDTA buffer (1mM, pH 8.5) for 5 min at 120°C in a pressure cooker. Ki67 [SP6] antibody (abcam, Cambridge, UK) at a dilution of 1:500 and murine ß-defensin-2 antibody (DEFB4 ABIN674574 from antibodies-online) at a dilution of 1:100 was applied for 30 min at room temperature. Subsequent reactions were performed with the Histofine Simple Stain Mouse MAX PO (rabbit) biotin-free horseradish peroxidase enzyme–labeled polymer detection system. Positive reactions were visualized with diaminobenzidine (DAB) (Dako) followed by counterstaining with hematoxylin (Merk, Darmstadt, Germany). Evaluation of the staining was performed semi-quantitatively by means of light microscopy (Carl Zeiss Vision, Hallbergmoos, Germany) in magnification as indicated.

### TUNEL assay

TUNEL assay on paraffin sections was conducted for the detection of apoptotic cells in CAP and placebo treated skin tissue according to [27] and was evaluated by fluorescence microscopy (Axio Imager Z1; Zeiss).

### Statistical analysis

Results are expressed as the mean ± SD. Comparisons between groups were made using Student’s unpaired t-test. A p value < 0.05 was considered statistically significant (*p < 0.05). All calculations were performed using the GraphPad Prism software package (GraphPad Software Inc., San Diego, CA, USA).

## Results

The aim of this study was to explore whether the CAP treatment using the MicroPlaSter ß affects gene expression and molecular mechanisms in differentiated skin keratinocytes in *in vitro* and *in vivo* approaches.

### CAP induces IL-8 expression in keratinocytes involved in inflammation, fibrosis and wound healing

Cytokines are extracellular signaling molecules that mediate cell-cell communication. They are released from cells and have critical roles in many biological processes such as immunity, inflammation, and fibrosis or wound healing. Amongst others, they regulate cellular growth, differentiation, gene expression, migration or cell proliferation [28–30]. In most biological processes, multiple cytokines operate in a large network, where the action of one cytokine is regulated by the presence or absence of other cytokines [30, 31].

In our previous study, we observed that different cytokines and acute phase proteins e.g. CD40 ligand, GRO alpha, IL-1ra, IL-6, IL-8, MCP-1, and Serpin E1 are positively affected by the CAP treatment in fibroblasts [20]. Hence, we were interested whether the CAP treatment targets the same molecules in keratinocytes or whether different cytokines are affected.

**Fig 1:**
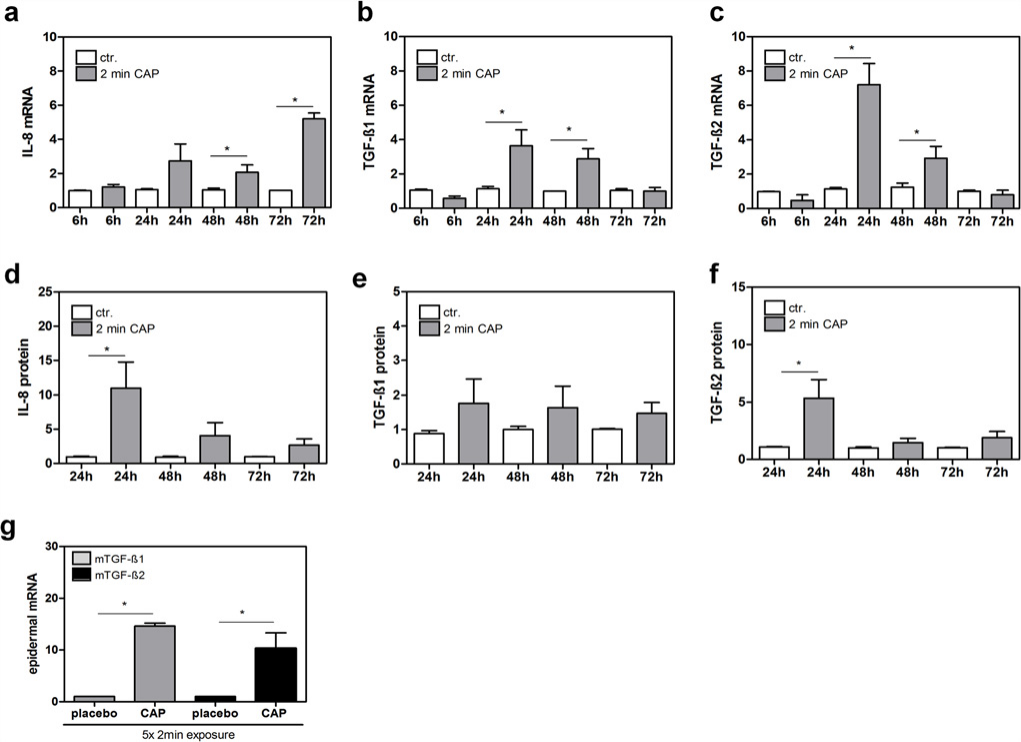
Expression of IL-8, TGF-ß1, and TGF-ß2 after the CAP treatment. (a, b, c) mRNA expression analysis of IL-8, TGF-ß1, and TGF-ß2 in human keratinocytes was performed 6 h, 24 h, 48 h, and 72 h after CAP treatment for 2 min by LightCycler 1.2 technology. (d, e, f) IL-8 secretion by human keratinocytes was analyzed 24 h, 48 h, and 72 h after CAP treatment for 2 min by FlowCytomix and TGF-ß1, and TGF-ß2 secretion by ELISA Technology (g) mRNA expression of murine TGF-ß1 (mTGF-ß1) and murine TGF-ß2 (mTGF-ß2) in epidermal skin was analyzed after 5x CAP treatment for 2 min and was compared to corresponding placebo control. *p < 0.05.

For analysis, keratinocytes were exposed to CAP for 2 min or remained untreated and were further incubated for 24 h before supernatants were collected and processed for the simultaneous detection of the relative expression levels of 36 human cytokines and acute phase proteins. Surprisingly, we observed that only interleukin 8 (IL-8) out of 36 analyzed cytokines was significantly up-regulated by the CAP treatment for 2 min (fold change (CAP/ctr.) 3.01; p value 0.0467). Array results concerning IL-8 induction were verified on mRNA level at different time points (6 h, 24 h, 48 h, and 72 h) after the CAP treatment (Fig. 1a) and on protein level determined by FlowCytomix Technology 24 h, 48 h, and 72 h after exposure (Fig. 1d). FlowCytomix results confirmed a significant IL-8 induction 24 h after CAP treatment. On mRNA level a significant induction was obtained after 48 h and 72 h, whereas a non-significant induction was already seen 24 h after CAP treatment. A dual effect of plasma could lead to a fast secretion of preformed IL-8 protein within 24 h on one hand and also to a production of new IL-8 on the other hand, which could explain the later IL-8 mRNA levels.

### CAP activates the expression of TGF-ß1 and TGF-ß2 in keratinocytes

TGF-ßs are the most studied molecules in wound healing and are well characterized in many inflammatory or fibrotic diseases. They are produced by several cell types including activated macrophages, fibroblasts, and keratinocytes and it is known that temporal activation of TGF-ßs promotes wound healing [32]. In addition, several studies demonstrated the relevance of TGF-ßs, since loss or over-expression are associated with wound healing defects or inflammatory diseases [33–36].

Therefore, we were interested whether the expression of TGF-ßs is modulated by the CAP treatment in keratinocytes. In analogy to our results obtained for fibroblasts [20], we observed that TGF-ß1 and TGF-ß2 are likewise induced in keratinocytes. On mRNA level, we observed a significant induction of TGF-ß1 24 h and 48 h after CAP treatment (Fig. 1b), whereas the induction of TGF-ß1 on protein level was not significant at the indicated time points (Fig. 1e). TGF-ß2 was significantly induced 24 h and 48 h after CAP treatment on mRNA level (Fig. 1c) and 24 h after treatment on protein level (Fig. 1f). Furthermore, both TGF-ßs are significantly up-regulated in the epidermal murine skin after 5x CAP treatments for 2 min compared to placebo control (Fig. 1g).

### Apoptosis is not affected by the CAP treatment in keratinocytes *in vitro* and *in vivo*

Different studies including our own study on malignant melanoma could show that apoptosis is induced in tumor cells by CAP treatment using different CAP devices and treatment conditions [37–39]. As described in the introduction part many studies observed apoptosis in normal cells after the CAP treatment [12–16, 40]. To ensure the safe usage of CAP on the skin, treatment parameters and CAP devices must be adapted to exclude/reduce apoptotic effects on normal cells. In our previous study we observed that the apoptotic machinery was not affected by the CAP treatment in fibroblasts under current clinical treatment conditions (2 min treatment using the MicroPlaSter ß) [20].

**Fig 2:**
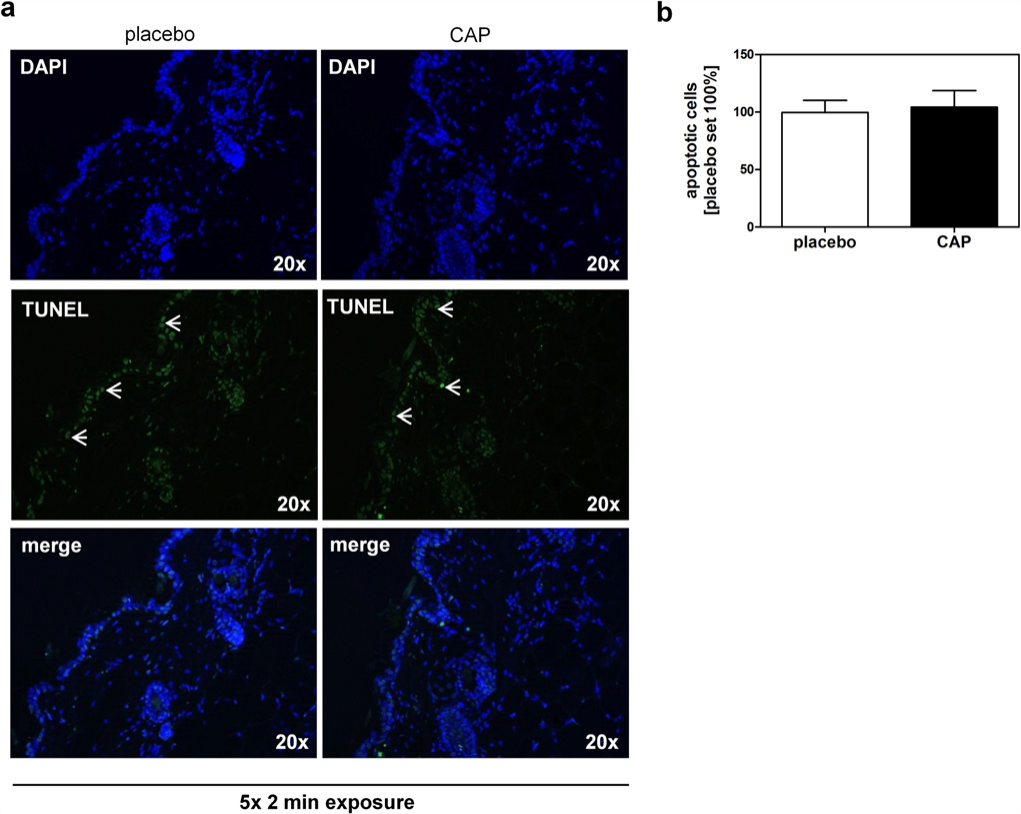
Detection of apoptotic cells in CAP and placebo treated murine skin tissue. Apoptotic cells were analyzed using a fluorometric TUNEL assay system for detection of fragmented DNA as indicator of apoptotic events. Representative photomicrographs are presented of placebo and CAP treated skin. Arrowheads exemplarily indicate apoptotic cells. Number of TUNEL positive cells were counted in three different fields and averaged. Placebo treatment was set to 100% ± SD. Magnification as indicated.

To analyze whether apoptosis is affected by CAP in keratinocytes, we used a human apoptosis array which detects 35 apoptosis-related proteins and searched for candidates differentially expressed 24 h after CAP treatment for 2 min. In analogy to our result obtained for fibroblasts [20] the evaluation of the array showed that these pro-apoptotic or anti-apoptotic factors were not influenced by the CAP treatment in keratinocytes (S1 Table). Additionally, we analyzed the skin of the treated mice for the appearance of apoptotic cells. Here, we observed that the number of apoptotic cells in the treated skin area did not differ between 5x CAP or 5x placebo treatment for 2 min (Fig. 2a, b). These results suggest that apoptotic mechanisms are not activated in keratinocytes by the CAP treatment under used treatment conditions.

### CAP treatment does not modulate keratinocyte proliferation and migration *in vitro* and *in vivo*

Next to apoptosis, cell viability is an important marker to screen for cytotoxic effects of CAP on eukaryotic cells. In order to determine the number of viable cells the XTT (2,3-Bis-(2-methoxy-4-nitro-5-sulfophenyl)-2H-tetrazolium-5-carboxanilide salt) assay was used.

**Fig 3:**
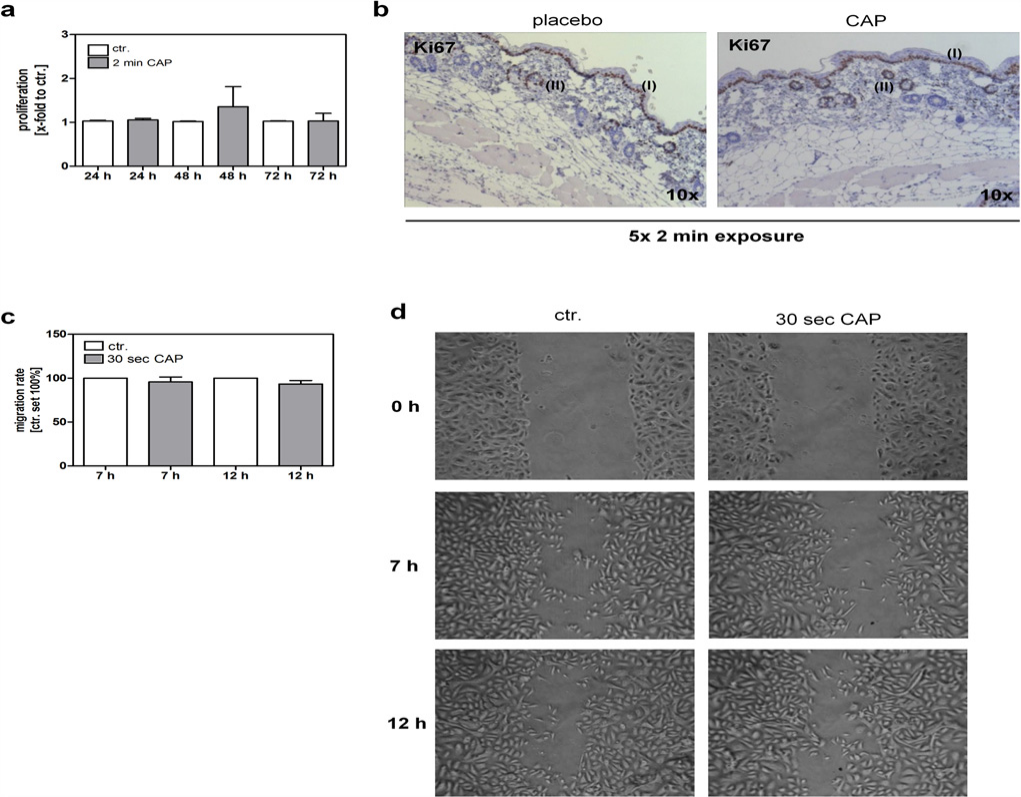
CAP effects on keratinocyte proliferation and migration. (**a**) Cell proliferation was determined using a XTT proliferation assay 24 h, 48 h and 72 h after the CAP exposure for 2 min and compared to control (ctr.). (**b**) Representative examples of immunohistochemical stains of formalin-fixed paraffin-embedded skin sections using Ki67, a proliferation marker that stains the nuclei of cells in G1, S, G2 and early mitosis. Highly proliferative, Ki67-positive keratinocytes were observed within the basal layer of the epidermis (I) and the bulge region of the hair follicle (II), whereas no difference between 5x placebo or 5x CAP treatment for 2 min was observed. Magnification as indicated. (**c, d**) Cell migration was determined using a wound healing assay. The migration rate was calculated 7 h and 12 h after culture insert was removed and displayed as the percentage relative to untreated control (ctr. set 100%). Representative images are shown immediately after culture insert was removed (0 h) and 7 h and 12 h later.

Proliferation of keratinocytes *in vitro* was not significantly changed after CAP treatment for 2 min analyzed 24 h, 48 h, and 72 h after exposure (Fig. 3a). Ki67 staining of skin sections confirmed our *in vitro* results, showing that the proliferation of cells was limited to keratinocytes of the basal layer of the epidermis (I) and the bulge region of the outer root sheath of the hair follicle (II) and did not differ between CAP and placebo control after 5x exposure for 2 min (Fig. 3b). Furthermore, we wanted to know whether migration is affected by the CAP treatment in keratinocytes because in our previous study we could show that a 30 sec treatment with CAP significantly activates fibroblast migration [20]. The treatment time for this assay has to be reduced because it is known that CAP treatment temporarily results in a detachment of the cells and inferential leads to a delayed migration ability [18]. In contrast to our results obtained for fibroblasts [20], we observed that migration was not significantly modified in keratinocytes treated with CAP for 30 sec (Fig. 3c, d).

### CAP promotes the expression of ß-defensins in keratinocytes

Based on the fact that CAP activates the pro-inflammatory cytokine IL-8 and the immunoreactive TGF-ß1 and TGF-ß2 in keratinocytes, we suggest that other inflammation-related molecules might be affected by CAP.

Further important inflammation-related molecules are the defensins. Defensins are small cysteine-rich cationic proteins and function as antimicrobial peptides (AMPs) [41, 42]. They are active against bacteria, fungi and many viruses. Cells of the immune system and almost all epithelial cells express these peptides to assist in killing phagocytosed bacteria. Most defensins function by binding to the microbial cell membrane, and, once embedded, forming pore-like membrane defects that allow efflux of essential ions and nutrients. Defensins are found in the human skin during inflammatory conditions like atopic dermatitis and also during wound healing [42, 43]. Since CAP is highly effective against bacteria we assumed that activation of antimicrobial peptides might contribute to the antiseptic efficacy of CAP observed *in vivo*.

**Fig 4:**
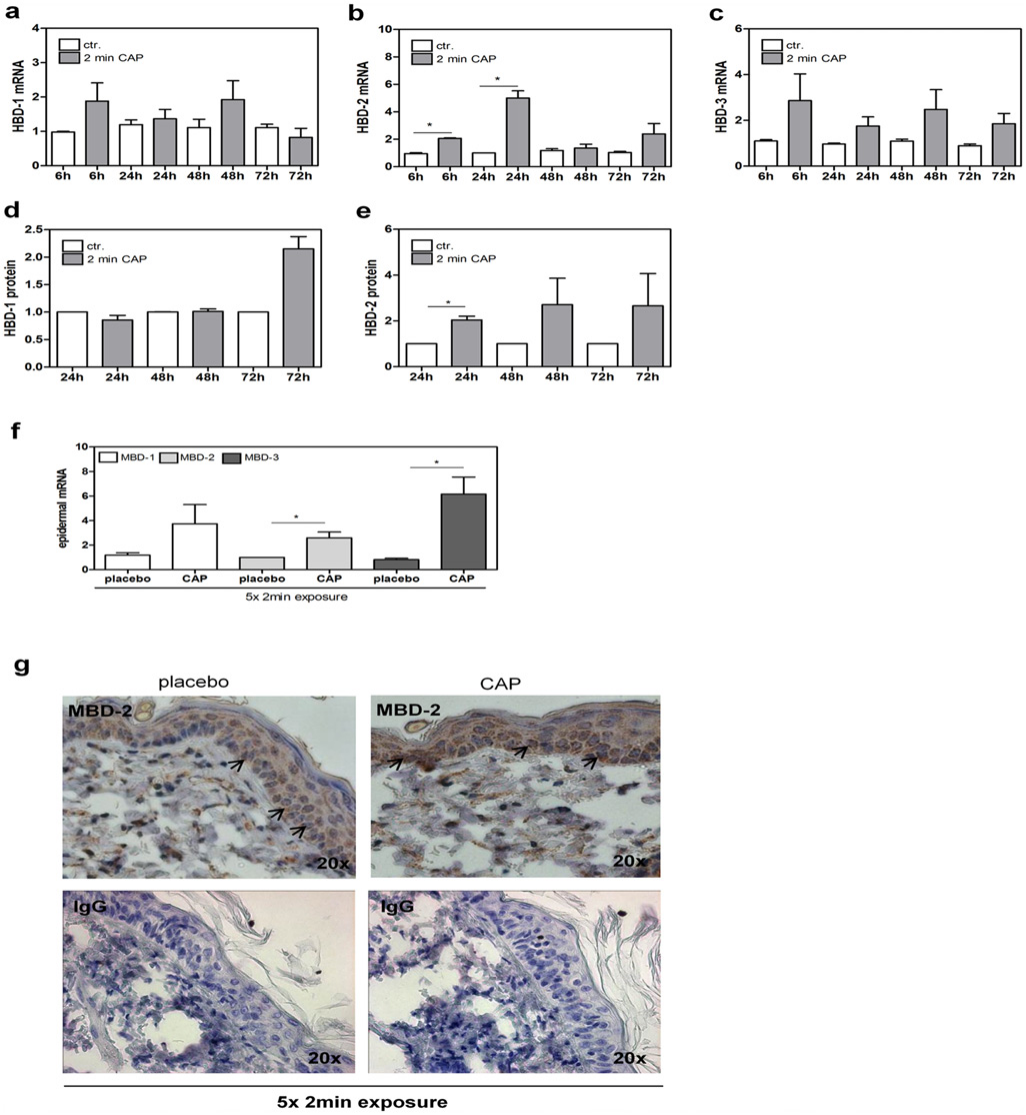
Human and murine ß-defensin expression after the CAP treatment. (a, b, c) mRNA expression of human ß-defensin-1 (HBD-1), ß-defensin-2 (HBD-2), and-3 (HBD-3) was analyzed 6 h, 24 h, 48 h, and 72 h in keratinocytes after the CAP treatment for 2 min. (**d, e**) ELISAs present the relative level of human BD-1 (HBD-1) and human BD-2 (HBD-2) secretion by keratinocytes after the CAP treatment for 2 min compared to control (ctr.). (**f**) mRNA expression of murine ß-defensins, MBD-1, MBD-2, and MBD-3 in epidermal skin tissue after 5x CAP treatment for 2 min compared to placebo control. *p < 0.05. (**g**) Immunohistochemistry against murine BD-2 (MBD-2) in murine tissue after 5x CAP treatment versus placebo control. Arrowheads exemplarily indicate MBD-2 positive keratinocytes, whereas a more intensive staining was observed in the CAP treated skin.

We analyzed the expression of human ß-defensin, HBD-1, HBD-2, and HBD-3, and observed that HBD-2 is significantly induced 6 h and 24 h after the CAP treatment for 2 min (Fig. 4b), whereas HBD-1 and HBD-3 mRNA expression was induced but not significantly by the CAP treatment (Fig. 4a, 4c). A significant induction of HBD-2 was also obtained on protein level 24 h after CAP treatment (Fig. 4e). The protein amount of HBD-1 was not significantly modified after CAP treatment for 2 min (Fig. 4d) and the sensitivity of the BD-3 ELISA was not sufficient to detect HBD-3 protein in the supernatant of both CAP and control treated keratinocytes (data no shown). These results let us speculate that under used treatment conditions HBD-2 is the most important human ß-defensin affected by the CAP treatment. Furthermore, also murine ß-defensins were analyzed in the epidermal skin after 5x CAP treatment for 2 min on mRNA level (Fig. 4f). MBD-2 and MBD-3 were significantly induced after the CAP treatment, whereas MBD-1 was not significantly induced. Immunohistochemistry confirmed an increased expression of murine MBD-2 in epidermal cells after the CAP treatment (Fig. 4g, arrowhead).

These data suggest that CAP promotes the expression of ß-defensins in human and murine keratinocytes and might contribute to the well-known antibacterial effects of the CAP treatment.

### CAP is able to directly activate gene expression in keratinocytes

Next, we wanted to distinguish whether CAP is able to directly affect gene expression in keratinocytes or whether the induction of genes is a result of a potential indirect activation of the innate immune system. In an *in vitro* approach, we incubated keratinocytes with cell culture supernatants obtained from keratinocytes directly treated with CAP for 2 min or from keratinocytes which remained untreated (ctr. supernatant) (S1 Fig.). The expression of IL-8, TGF-ß1, TGF-ß2, HBD-1, and HBD-2 in keratinocytes exposed to supernatants of CAP-treated keratinocytes was not significantly modified, suggesting that CAP primarily affects these genes directly. Interestingly, HBD-2 was significantly induced 72 h after keratinocytes had been exposed to CAP supernatants. Here, we cannot exclude any kind of indirect CAP induction.

In an *in vivo* approach, we tried to circumvent any potential indirect activation of the innate immune system by using an antiseptic treatment prior to the use of CAP or placebo treatment. A non-significant induction of TGF-ß1 and a significant induction of TGF-ß2 were obtained in the murine epidermal skin when we performed an antiseptic treatment with Octenisept prior to the CAP treatment (S2a Fig.). Furthermore, also MBD-1 and MBD-3 were significantly induced under these treatment conditions (S2b Fig.), suggesting that CAP is able to directly activate gene expression in keratinocytes.

## Discussion

Over the past few years, plasma medicine has become an important field in medical science. Cold atmospheric plasma (CAP) has proven anti-inflammatory, anti-microbial and anti-neoplastic effects and promotes wound healing [5, 44, 45]. Before implementing plasma as new medical treatment tool, effects and safety on eukaryotic cells must be proven.

In the present study we aimed to define differentially regulated genes and molecular mechanisms, which are affected by the CAP treatment. Keratinocytes are in focus of the present work, because these cells are primarily affected by skin disinfection or skin/wound treatments and concerning plasma effects they are not sufficiently analyzed so far.

Interestingly, we observed that analyzed cytokines and acute phase proteins, crucial for inflammation, fibrosis or wound healing, were practically not affected by CAP in keratinocytes under used treatment conditions (2 min treatment using the MicroPlaSter ß). Only the pro-inflammatory cytokine IL-8 was significantly up-regulated *in vitro*. IL-8 can be induced directly or indirectly by the CAP treatment. It might be possible that after CAP treatment of 2 min single cells are damaged or are temporarily detached from the cell culture dish and are able to induce parts of the innate immune system of surrounding keratinocytes in an autocrine mode. At the other hand it is known from the literature that IL-8 is constitutively expressed in normal cultured keratinocytes and increases rapidly after UVB irradiation [46], which is also directly discharged by CAP. To analyze, whether CAP affects keratinocytes directly or indirectly, we incubated keratinocytes with supernatants obtained from CAP-treated keratinocytes. Interestingly, we observed no significant induction of IL-8, TGF-ß1, TGF-ß2, HBD-1, and HBD-3 (S1 Fig.), suggesting that CAP predominantly affects gene expression in these cells directly. The significant induction of HBD-2 observed 72 h after incubation with CAP-treated supernatants indicates that indirect CAP effects may also exist.

Since CAP is able to kill bacteria, it would be possible that this antimicrobial mechanism can indirectly affect the induction of these genes. To analyze this we performed an antiseptic treatment prior the use of CAP or placebo treatment in our mouse model to exclude antimicrobial influences. The significant induction of murine TGF-ß2, MBD-1, and MBD-3 on mRNA level (S2 Fig.) support that CAP directly affects gene expression in keratinocytes. Interestingly, the induction intensity of all analyzed genes was lower using an antiseptic treatment prior to each CAP treatment in comparison to the CAP treatment alone (Fig. 1g and Fig. 4f), suggesting that antimicrobial influences may also exist, which must be investigated in future research. Additionally, paracrine mechanisms must be also considered *in vivo*. A possibility why nearly no cytokine, chemokine, and acute phase protein is regulated by the CAP treatment in keratinocytes might be the absence of paracrine mechanisms in our *in vitro* approaches. During wound healing we observed that CAP induces different cytokines in fibroblasts *in vitro* as well as *in vivo* [20], whereas these molecules are not affected in keratinocytes. We believe that the interplay between fibroblasts and keratinocytes is required to provoke cellular mechanisms important for e.g. wound healing.

Remarkable, however, is the activation of TGF-ßs after CAP treatment. Our results from this work and our previous study [20] revealed that TGF-ß1 and TGF-ß2 are induced in keratinocytes as well as in fibroblasts after the CAP treatment. It is known that TGF-ß can both act on and be secreted by diverse cell types which participate in the wound healing process. Actions of TGF-ß to stimulate chemotaxis, fibrogenesis, angiogenesis, and autoinduction of its expression have shown to improve healing in a variety of animal models of both normal and impaired wound healing [47].

In our study we observed that apoptosis is not provoked by CAP under used conditions. Furthermore, we observed that cell viability (analyzed by XTT proliferation assay) *in vitro* and cell proliferation (analyzed by Ki67 immunohistochemical staining) *in vivo* was also not modified under applied conditions in keratinocytes. These results are desirable and fulfill basic recommendations for the safe use of the MicroPlaSter ß on the skin. Important to note is that we observed an accelerated re-epithelialization (due to an increased keratinocyte proliferation) after CAP treatment in an animal wound healing model [20]. We suggest that the CAP discrepancy concerning cell proliferation depends on the property of the skin and varies between healthy and wounded skin. Regrettably, the complex mechanism and the molecular changes which may elucidate the present phenomenon are not investigated so far.

To ensure the safe usage of CAP on the skin (e.g., for skin disinfection, for wound treatment or treatment of skin diseases) further studies concerning mutagenicity and cancerogenicity must be performed. Own unpublished data and results from Boxhammer and colleagues [48] suggest that mutagenicity is presumably not inducible in eukaryotic cells under used treatment conditions, whereas further studies using different cell types, plasma devices, and treatment conditions must approve this suggestion.

Several studies revealed that plasma exhibits strong antimicrobial efficacy *in vitro* and *in vivo* by directly killing bacteria [4, 6–9, 49]. Our present study describes an absolutely new plasma strategy in order to reduce the bacterial load: the activation of defensins in keratinocytes. Defensins are host-defense peptides produced by eukaryotic cells which perform antimicrobial functions to combat infection during wound healing (Gibson et al., 2012) or other skin diseases [43, 50, 51]. We observed that different human and murine ß-defensins are inducible by the CAP treatment in keratinocytes. Future studies must clarify how CAP exactly activates ß-defensins in keratinocytes because induction of host defense peptides is an important mechanism requested for skin disinfection or chronic wound treatment.

In summary, this study provides evidence that a short application of CAP using the MicroPlaSter ß does not cause apoptosis and has no impact on keratinocyte migration and proliferation *in vitro* or on healthy skin. Furthermore, we observed that CAP activates human and murine antimicrobial peptides in keratinocytes, which support the well-known antibacterial effects of CAP treatment.

## Supporting Information

S1 Fig Effect on gene expression after indirect CAP treatment *in vitro*. (**a-f**) mRNA expression of human IL-8, TGF-ß1, TGF-ß2, HBD-1, HBD-2, and HBD-3 in keratinocytes treated with supernatants obtained from keratinocytes directly affected by CAP or control treatment for 2 min. The supernatants were obtained 24 h after exposure and remained on the cells as indicated (6 h, 24 h, 48 h, 72 h). *p < 0.05.
(TIF)

S2 Fig Effect on gene expression after an antiseptic treatment prior to the CAP treatment *in vivo*. Expression of (**a**) murine TGF-ßs (mTGF-ß1 and mTGF-ß2) and (**b**) murine ß-defensins (MBD-1, MBD-2, and MBD-3) in epidermal skin after a standard antiseptic Octenisept treatment prior to a 2 min CAP or placebo treatment, 5 days long. *p < 0.05.
(TIF)

S1 Table Protein Array results. Fold change (CAP/control) and p value of analyzed molecules from Human Apoptosis Antibody Array (R&D Systems,Inc.). *p<0.05; ns: not significant.
(TIF)
