# Supplementary figures and images for: Effects of Cold Atmospheric Plasma (CAP) on ß-Defensins, Inflammatory Cytokines, and Apoptosis-Related Molecules in Keratinocytes In Vitro and In Vivo
Source: PLoS One. 2015 Mar 13;10(3):e0120041. doi: 10.1371/journal.pone.0120041 (PMC4359157; doi:10.1371/journal.pone.0120041)

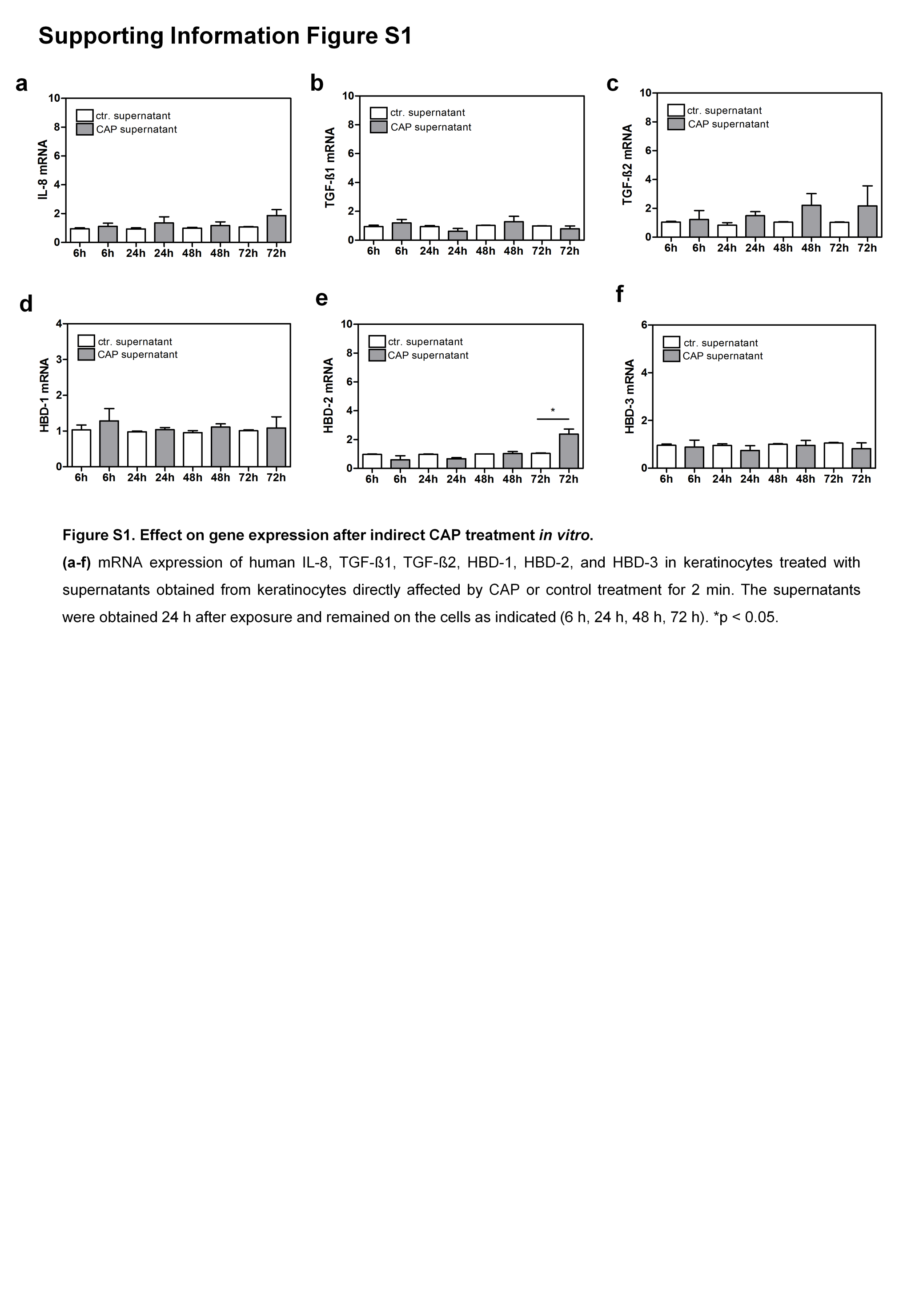

Supplement: S1 Fig — (a-f) mRNA expression of human IL-8, TGF-ß1, TGF-ß2, HBD-1, HBD-2, and HBD-3 in keratinocytes treated with supernatants obtained from keratinocytes directly affected by CAP or control treatment for 2 min. The supernatants were obtained 24 h after exposure and remained on the cells as indicated (6 h, 24 h, 48 h, 72 h). *p < 0.05. (TIF) [file pone.0120041.s001.tif]

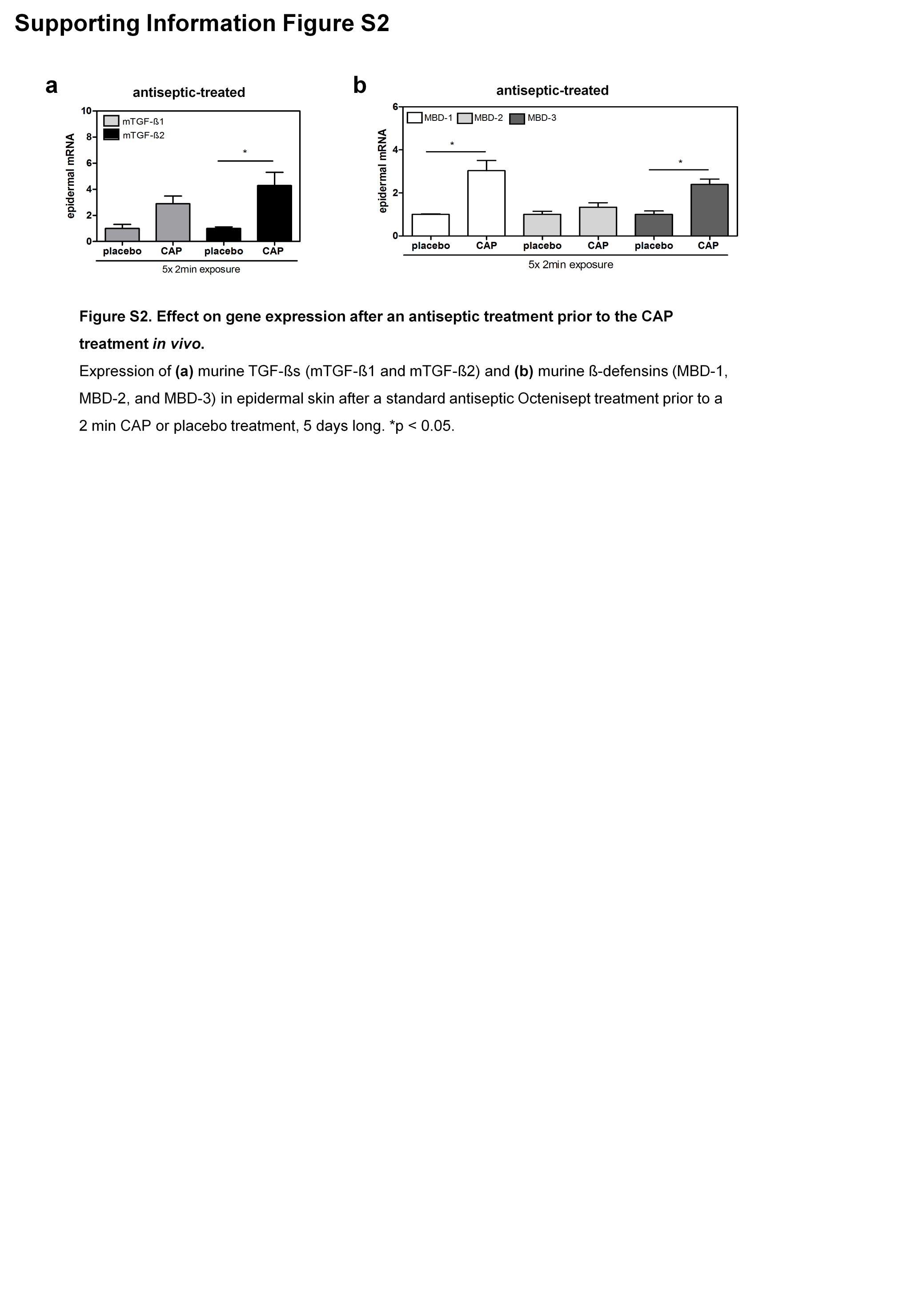

Supplement: S2 Fig — Expression of (a) murine TGF-ßs (mTGF-ß1 and mTGF-ß2) and (b) murine ß-defensins (MBD-1, MBD-2, and MBD-3) in epidermal skin after a standard antiseptic Octenisept treatment prior to a 2 min CAP or placebo treatment, 5 days long. *p < 0.05. (TIF) [file pone.0120041.s002.tif]

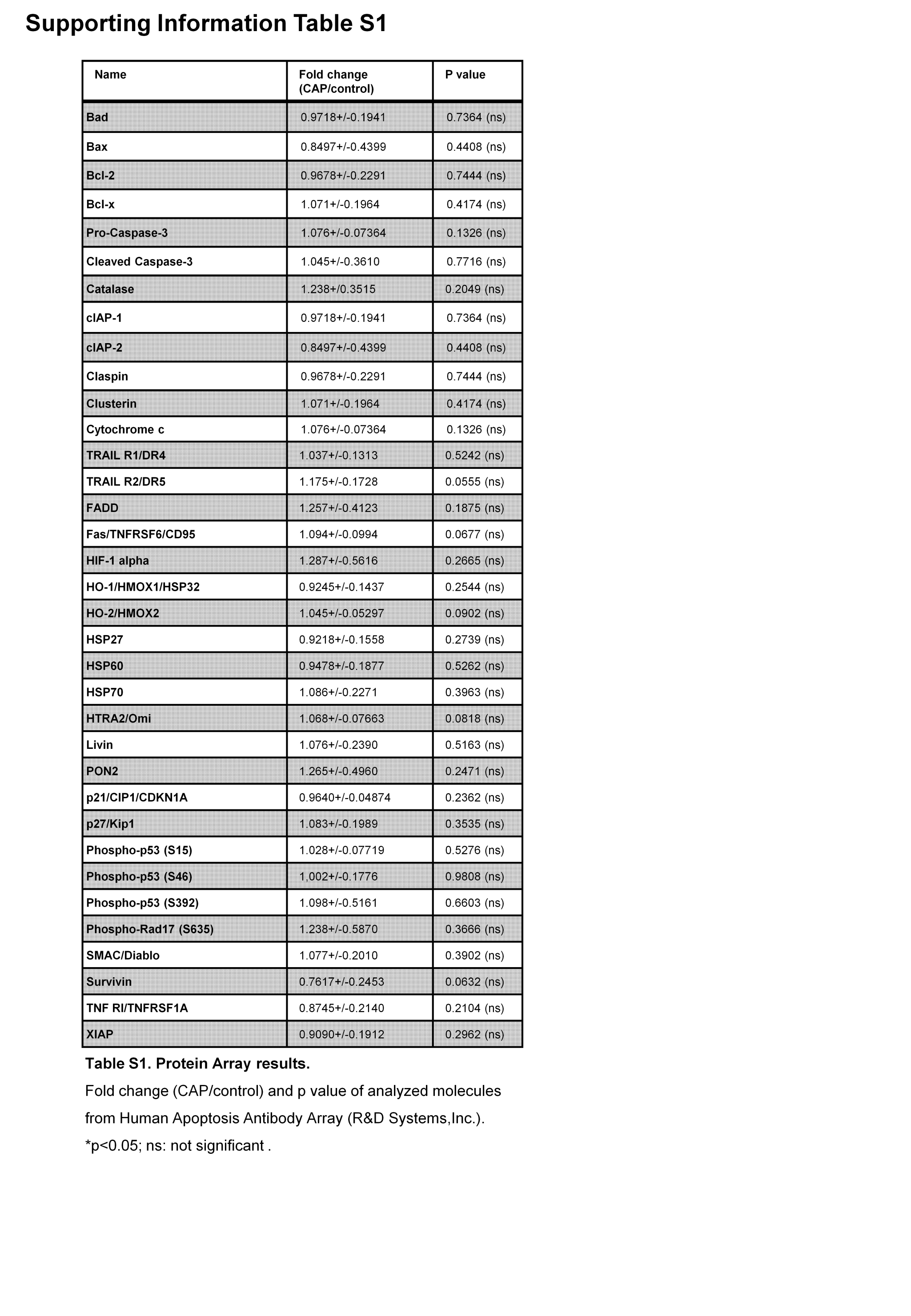

Supplement: S1 Table — Fold change (CAP/control) and p value of analyzed molecules from Human Apoptosis Antibody Array (R&D Systems,Inc.). *p<0.05; ns: not significant. (TIF) [file pone.0120041.s003.tif]
